# YKT6 Is Essential for Male Fertility by Promoting Meiosis Progression During Spermatogenesis of Mice

**DOI:** 10.1111/cpr.70079

**Published:** 2025-06-18

**Authors:** Jie Cen, Xiaochen Yu, Ziqi Wang, Wenbo Liu, Jianze Xu, Qian Fang, Fei Gao, Yongzhi Cao, Hongbin Liu

**Affiliations:** ^1^ Institute of Women, Children and Reproductive Health Shandong University China; ^2^ State Key Laboratory of Reproductive Medicine and Offspring Health Shandong University Jinan Shandong China; ^3^ National Research Center for Assisted Reproductive Technology and Reproductive Genetics Shandong University Jinan Shandong China; ^4^ Key Laboratory of Reproductive Endocrinology (Shandong University) Ministry of Education Jinan Shandong China; ^5^ Shandong Technology Innovation Center for Reproductive Health Jinan Shandong China; ^6^ Shandong Provincial Clinical Research Center for Reproductive Health Jinan Shandong China; ^7^ Center for Reproductive Medicine The First Affiliated Hospital, Zhejiang University School of Medicine Hangzhou China; ^8^ Department of Ultrasound Qilu Hospital of Shandong University Jinan Shandong China; ^9^ State Key Laboratory of Stem Cell and Reproductive Biology, Institute of Zoology Chinese Academy of Sciences Beijing China; ^10^ Model Animal Research Center Shandong University Jinan Shandong China; ^11^ CUHK‐SDU Joint Laboratory on Reproductive Genetics, School of Biomedical Sciences The Chinese University of Hong Kong Hong Kong China

**Keywords:** intercellular bridge, meiotic arrest, spermatogenesis, vesicular transport, YKT6

## Abstract

SNARE proteins are required for membrane fusion events throughout the endomembrane system, and are therefore associated with vesicular transport. Here, we found that the SNARE family member, YKT6, is indispensable for male fertility in mice. Conditional *Ykt6* knockout in pre‐meiotic and meiotic germ cells leads to complete sterility and meiotic arrest in male mice, which exhibit loss of spermatocytes in seminiferous tubules, but without obvious disruption of chromosomal behaviours during meiosis. We observed that the abundance of syncytia increases along with abnormal morphology of the Golgi apparatus, while lysosomes decrease in *Ykt6*‐cKO testes. Quantitative proteomics and immunofluorescent staining both showed dysregulation of vesicular transport in YKT6‐deficient spermatocytes. Additionally, the recombinant mouse proteins, HA::YKT6 and MYC::STX1A, could interact in vitro, further supporting a likely role in mediating transport vesicle fusion with the plasma membrane. Finally, the absence of TEX14 signal within syncytia and enlarged TEX14 rings between spermatocytes together suggest a failure to stabilise intercellular bridges in *Ykt6*‐cKO testes. These results demonstrate that YKT6 is required for male fertility by promoting meiosis progression through vesicular transport regulation during spermatogenesis in mice, expanding our understanding of YKT6 functions, and suggesting a possible strategy for future interventions for male infertility in humans.

## Graphical abstract

Specifically ablating *Ykt6* in pre‐meiotic germ cells and meiotic spermatocytes leads to complete sterility and meiotic arrest in conditional knockout male mice. YTK6 is required for stabilising intercellular bridges between spermatocytes due to its key role in maintaining protein homeostasis and vesicular transport‐dependent functions in spermatocytes, without impeding meiotic chromosomal behaviours.

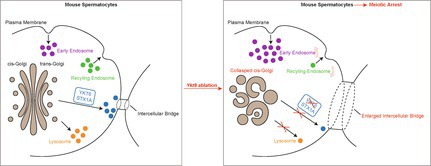

## 1 Introduction

Spermatogenesis is a complex and tightly orchestrated process that includes mitotic amplification of spermatogonia, meiosis of spermatocytes and finally post‐meiotic spermiogenesis [1, 2]. Meiosis is a specialised process of cell division through which genetic material is duplicated once, after which cells divide twice in succession, resulting in gametes with half the number of chromosomes [3]. Based on morphological changes in chromosomes, meiotic prophase I can be divided into the leptotene, zygotene, pachytene, diplotene and diakinesis stages [4, 5]. To ensure the proper separation of homologous paternal chromosomes in meiosis, cells undergo a tightly coordinated series of events, beginning with the formation of programmed DNA double‐stranded breaks (DSBs) in homologous chromosomes at the leptotene stage. DSB formation triggers the recruitment of meiosis‐related factors that subsequently initiate homologous recombination to repair DNA damage [6]. During the zygotene stage, recombination‐related proteins are recruited to the site of meiotic DSBs, leading to the formation of so‐called recombination foci that facilitate synapsis of homologous chromosomes [7]. When the chromosomes are fully synapsed at the pachytene stage, the recombination foci continue to mature, finally resolving into crossovers [8]. Homologous chromosomes then dissociate at the diplotene stage, and the synaptonemal complex begins to disassemble [9]. The formation and repair of DSBs, crossover recombination and assembly/disassembly of the synaptonemal complex thus represent key events in meiosis. Defects at any stage in meiosis prophase I typically lead to meiotic arrest and sterility [7]. During spermatogenesis, germ cells undergo an incomplete cytoplasmic division. At the termination of cytokinesis, somatic cells are connected transiently by an intercellular bridge preceding abscission. In germ cells, these transient structures are instead transformed into stable intercellular bridges, allowing synchronisation via the passage of organelles and molecules between germ cells without abscission, which is crucial for germ cell viability, and consequently, male fertility [10, 11, 12].

Eukaryotic cells are highly compartmentalised with membrane‐enclosed intracellular organelles that communicate with each other through the exchange of trafficking vesicles [13]. Precise protein and lipid transport between organelles, driven primarily by vesicle‐mediated membrane trafficking through the secretory and endocytic pathways, typically involves the generation of a vesicle from a precursor membrane, subsequent transport of the vesicle to its destination, and finally ending in vesicle fusion with the target compartment [14]. Soluble N‐ethylmaleimide‐sensitive factor attachment protein receptors (SNAREs) are a family of small, conserved, eukaryotic proteins that mediate this process of membrane fusion between organelles and the plasma membrane [15]. For membrane fusion to occur, SNAREs on opposing membranes must come together so that their respective SNARE motifs can interact through a zippering process that results in the formation of a SNARE complex [15]. SNAREs are required for membrane fusion throughout the endomembrane system, and are therefore commonly associated with vesicular transport [16].

Among these SNARE proteins, YKT6 lacks a transmembrane domain but instead carries post‐translational modifications, including farnesylation and palmitoylation, that control its membrane association and fusogenic activity [17]. YKT6 is highly conserved between yeast and humans and has emerged as a key protein in a wide array of trafficking processes, such as ER‐Golgi transport, intra‐Golgi trafficking, endosome‐Golgi transport, autophagosome formation and constitutive secretion to the plasma membrane [16, 17, 18, 19, 20, 21, 22, 23, 24]. Additionally, YKT6 has been implicated in a number of human diseases, including Parkinson's disease, developmental delay with or without severe infantile liver disease and risk of hepatocellular carcinoma [25, 26, 27]. Although a previous study has shown that YKT6 plays an important role in oogenesis in *Drosophila* [28], its possible role in spermatogenesis remains unknown.

To explore its possible function in male fertility in our present study, we generated mice with conditional *Ykt6* knockout (*Ykt6*‐cKO) in pre‐meiotic and meiotic male germ cells, which resulted in male sterility and meiotic arrest, but without abnormal meiotic chromosomal behaviours. *Ykt6*‐cKO testes showed increased syncytia numbers, with a collapsed morphology of the cis‐Golgi apparatus and sparse lysosome formation in spermatocytes. Multiple lines of evidence suggested disruption of vesicular transport, which was further supported by in vitro assays showing that YKT6 could interact with Syntaxin 1A (STX1A), which is required for transport vesicle fusion to the plasma membrane. Finally, the lack of Testis‐expressed gene 14 (TEX14) signal within syncytia and increased diameter of TEX14 rings between spermatocytes imply that YKT6 potentially plays a role in stabilising intercellular bridges during meiosis and further supports a scenario in which YKT6 is required to promote meiosis progression through regulation of vesicular transport in spermatocytes. This study thus uncovers a previously unrecognised SNARE required for meiosis prophase I in spermatogenesis and expands the scope of our understanding of SNARE family function in reproduction.

## 2 Materials and Methods

### 2.1 Mice

The care and breeding of mice, and all animal experiments, were approved by and conducted according to the guidelines of the Animal Ethics Committee of the School of Medicine, Shandong University (Jinan, Shandong Province, China). All mouse models were generated in the C57BL/6J genetic background and maintained under specific pathogen‐free (SPF) conditions with free access to water and food and daily cycles of 12 h dark/12 h light.

*Ykt6* ^*flox/flox*^ mice were generated by GemPharmatech Co. (Jiangsu, China) using a CRISPR‐Cas9‐based gene editing approach. According to the structure of the *Ykt6* gene, exon3‐exon6 of *Ykt6‐201* (ENSMUST00000002818.8) transcript was selected as the knockout region. In brief, sgRNAs were transcribed in vitro, and the donor vector was constructed. Cas9, sgRNA and the donor were microinjected into fertilised eggs of C57BL/6J mice. The fertilised eggs were then transplanted to obtain positive F0 mice, which were confirmed by PCR and sequencing. Stable F1 generation mice were obtained by mating positive F0 mice with C57BL/6J mice. To construct germ cell‐specific *Ykt6* knock out mice, *Ykt6* ^*flox/flox*^ mice were mated with *Stra8‐GFPCre* mice generously gifted by Prof. Ming‐han Tong at the Chinese Academy of Sciences.

### 2.2 Antibodies

The antibodies for Western blotting targeted YKT6 (Abcam, ab241382), GAPDH (Proteintech, 10,494‐1‐AP), LAMIN B1 (Proteintech, 12,987‐1‐AP), β‐ACTIN (Proteintech, 66,009‐1‐Ig), TOGLN2 (Abcam, ab16059), GM130 (BD Biosciences, 610,822), LAMP2 (Abcam, ab13524), HA (Cell Signalling Technology, 3724) or MYC (Cell Signalling Technology, 2276). Antibodies against PNA (Invitrogen, L21409), DAPI (Abcam, ab104139), pH 3 (Cell Signalling Technology, 9701), SYCP3 (Abcam, ab15093, ab97672), γH2AX (Millipore, 05–636), MLH1 (BD Biosciences, 550,838), SYCP1 (Abcam, ab15090), Na/K ATPase (Abcam, ab76020), GM130 (BD Biosciences, 610,822), TOGLN2 (Abcam, ab16059), LAMP2 (Abcam, ab13524), RAB5 (Cell Signalling Technology, 3547), RAB11 (Cell Signalling Technology, 5589), STX1A (Abcam, ab272736) or TEX14 (Proteintech, 18,351‐1‐AP) were used for immunofluorescent staining. Primary antibodies were detected with Alexa Fluor 488‐ or 594‐conjugated secondary antibodies (Abcam, ab150077, ab150120, ab150080, ab150117). The antibodies for immunoprecipitation targeted HA (Cell Signalling Technology, 3724) or MYC (Cell Signalling Technology, 2276).

### 2.3 Western Blots

To prepare protein extracts, testis extracts were prepared using a homogeniser in RIPA buffer (Beyotime Biotechnology, P0013B) supplemented with 100 mM PMSF (Beyotime Biotechnology, ST506). After homogenization, the testes lysates were incubated on ice for 30 min and then centrifuged at 4°C, 13000 × g for 20 min. Supernatants were transferred to new tubes and 5 × SDS‐PAGE protein loading buffer (Beyotime Biotechnology, P0015L) was added. After boiling at 95°C for 10 min, the protein lysates were used for western blots.

### 2.4 Fertility Test of Male Mice

All *Ykt6*‐Ctrl and *Ykt6*‐cKO male mice (8–12 weeks, *n* = 6) were caged with two 8‐week‐old wild‐type C57BL/6J females in the evening, and vaginal plugs were checked every morning. Once the plug was observed (0.5 days after mating), the female mice were separated and individually housed, while the male mice were allowed a 2‐day rest period before another round of mating with another two females. The plugged females were separated and monitored for their pregnancy status and litter size. The average number of pups per litter was documented, and the fertility test lasted for at least 4 weeks.

### 2.5 Histology, Immunostaining and TUNEL Analysis of Paraffin Sections

Testes and epididymides dissected from mice were fixed with Bouin's solution (Sigma‐Aldrich, HT10132) or 4% paraformaldehyde (PFA) (Solarbio, P1110) at 4°C overnight for histological or immunostaining analysis, respectively. Human testis sections were testis biopsy specimens from a patient with congenital bilateral absence of the vas deferens but normal sperm production, approved by the Ethics Committee of Reproductive Hospital Affiliated to Shandong University. The samples were dehydrated, embedded in paraffin and cut into 5 μm sections. Sections were then stained with haematoxylin for histological analysis and analysed with an epifluorescence microscope (BX52, Olympus). For immunofluorescence analysis, sections were boiled in 10 mM sodium citrate buffer (pH 6.0) for 20 min. After soaking in PBS containing 0.1% tritonX‐100 (PBST) for 10 min, sections were blocked with 5% bovine serum albumin (BSA) for one hour in a humidity chamber. The primary antibodies were incubated overnight at 4°C. After washing in PBS, the slides were incubated in Alexa‐conjugated secondary antibodies at room temperature for one hour. Slides were mounted with coverslips using a mounting medium with DAPI (Abcam, ab104139). TUNEL assays were strictly carried out according to the manufacturer's instructions accompanying the One Step TUNEL Apoptosis Assay Kit (Meilunbio, MA0223). Images were obtained by confocal microscopy using an Andor Dragonfly spinning disc confocal microscope driven by Fusion Software, and projection images were prepared using Bitplane Imaris software.

### 2.6 Immunostaining of Spermatocytes and Fluorescence‐Activated Cell Sorting

After removing the tunica albuginea, testes were dispersed lightly with tweezers, and the seminiferous tubules were incubated in 1× collagenase type I (120 U/mL, Thermo Fisher Scientific, 17,100,017) at 35°C with gentle rotation for 10 min. After further digestion with 5 mL 0.25% trypsin containing 5 mg/mL DNase I for 7 min at 35°C with rotation, tubule fragments were pipetted up and down using straws to disperse germ cells. Trypsin was inactivated with 0.5 mL of fetal bovine serum (FBS) and the suspension was filtered through 70 μm honeycomb filters. The suspension was centrifuged and resuscitated in PBS containing 4% PFA for immunostaining or in DMEM containing 10% FBS for fluorescence‐activated cell sorting (FACS).

For immunofluorescence analysis, cell suspensions were loaded onto slides and dried in a humid chamber at room temperature for 4–5 h. After soaking in PBST for 10 min, sections were blocked with 5% BSA for one hour. The primary antibodies were incubated overnight at 4°C. After washing in PBS, the slides were incubated in Alexa‐conjugated secondary antibodies at room temperature for one hour. Slides were mounted with coverslips using a mounting medium with DAPI. For FACS, the cell suspensions were stained with Hoechst 33342 (1 × 106/ml, 3 μg/μL) at 35°C for 40 min. Then, the cells were sorted by flow cytometer (BD Biosciences, FACS Aria II, USA).

### 2.7 Spermatocyte Chromosome Spreads

Testes were dissected and seminiferous tubules were incubated in hypotonic extraction buffer (30 mM Tris, 50 mM sucrose, 17 mM trisodium citrate, 5 mM ethylenediaminetetraacetic acid, 0.5 mM dithiothreitol; adjusted to pH 8.2) for 30–40 min at room temperature. Subsequently, the tubules were shredded between the tips of two fine watchmaker forceps in 100 mM sucrose with pH 8.2, and the cell suspensions were loaded onto slides containing fixation solution (1% PFA and 0.15% Triton X‐100, pH 9.2). The slides were dried in a humid chamber at room temperature for 4–5 h and stored at −80°C.

### 2.8 Immunoprecipitation

Full‐length cDNAs encoding YKT6 were cloned into the pCDNA3.1–3 × HA‐C mammalian expression vectors (MiaoLing Plasmid Platform, Wuhan, China). Full‐length cDNAs encoding STX1A were cloned into the pCDNA3.1–3 × Myc‐C mammalian expression vectors (MiaoLing Plasmid Platform, Wuhan, China). pCDNA3.1–3 × HA‐C and pCDNA3.1–3 × Myc‐C vectors were used as negative controls. HEK 293 T cells were transiently transfected using XtremeGENE HP DNA Transfection Reagent (Roche). Transfected cells were lysed in TAP lysis buffer (50 mM HEPES‐KOH, pH 7.5, 100 mM KCl, 2 mM EDTA, 10% glycerol, 0.1% NP‐40, 10 mM NaF, 0.25 mM Na3VO4,50 mM β‐glycerolphosphate) plus protease inhibitors for 30 min on ice, then centrifuged at 13000 × g for 15 min. The antibody was added and the mixture was rotated in Eppendorf tubes at 4°C overnight. The resulting immunocomplexes were isolated by adsorption to protein A/G Sepharose beads for one hour. After washing, the beads were loaded onto 4%–20% Tris‐Glycine Mini Gels (Invitrogen) and separated proteins were detected by Western blots with the indicated antibodies.

### 2.9 Proteomics Analysis

The tetraploid spermatocytes obtained from *Ykt6*‐Ctrl and *Ykt6*‐cKO mice at postnatal days (PD) 16 by fluorescence‐activated cell sorting were subjected to proteomics analysis. After three rinses with phosphate‐buffered saline (PBS), the samples were gathered into tubes containing lysis buffer, flash‐frozen with liquid nitrogen and transferred to a −80°C freezer. The samples were transported on dry ice to the National Center for Protein Sciences at Peking University, Beijing, where they underwent quantitative proteomic determination.

The principal components analysis (PCA) plot was generated using R package ggplot2 [29], and the Venn diagram was generated using R package VennDiagram [30]. The differential expression analysis was carried out to identify proteins that exhibited significantly differential abundance in comparisons between *Ykt6*‐Ctrl and *Ykt6*‐cKO samples by using R package DESeq2 [31] with the following screening criteria: |log_2_‐fold‐change| (|log_2_FC|) > 1.0 and *p* < 0.05, and then visualised using R package ggpubr [32]. GO (Gene Ontology) analysis and Gene set enrichment analysis (GSEA) were carried out using R package clusterProfiler [33] and a threshold of *p* < 0.05 was applied for the analyses. The whole process was performed using Hiplot Pro (https://hiplot.com.cn/), a comprehensive web service for biomedical data analysis and visualisation.

### 2.10 Statistical Analysis

Unless stated otherwise, all experiments and statistical analyses in this study were performed with at least three independent replicates. Significance tests and correlation analyses were conducted using GraphPad Software, and significant differences between groups were determined by two‐tailed unpaired Student's t‐tests, with significance defined by *p < 0.05.

## 3 Results

### 3.1 YKT6 Is Required for Spermatogenesis and Male Fertility

**FIGURE 1:**
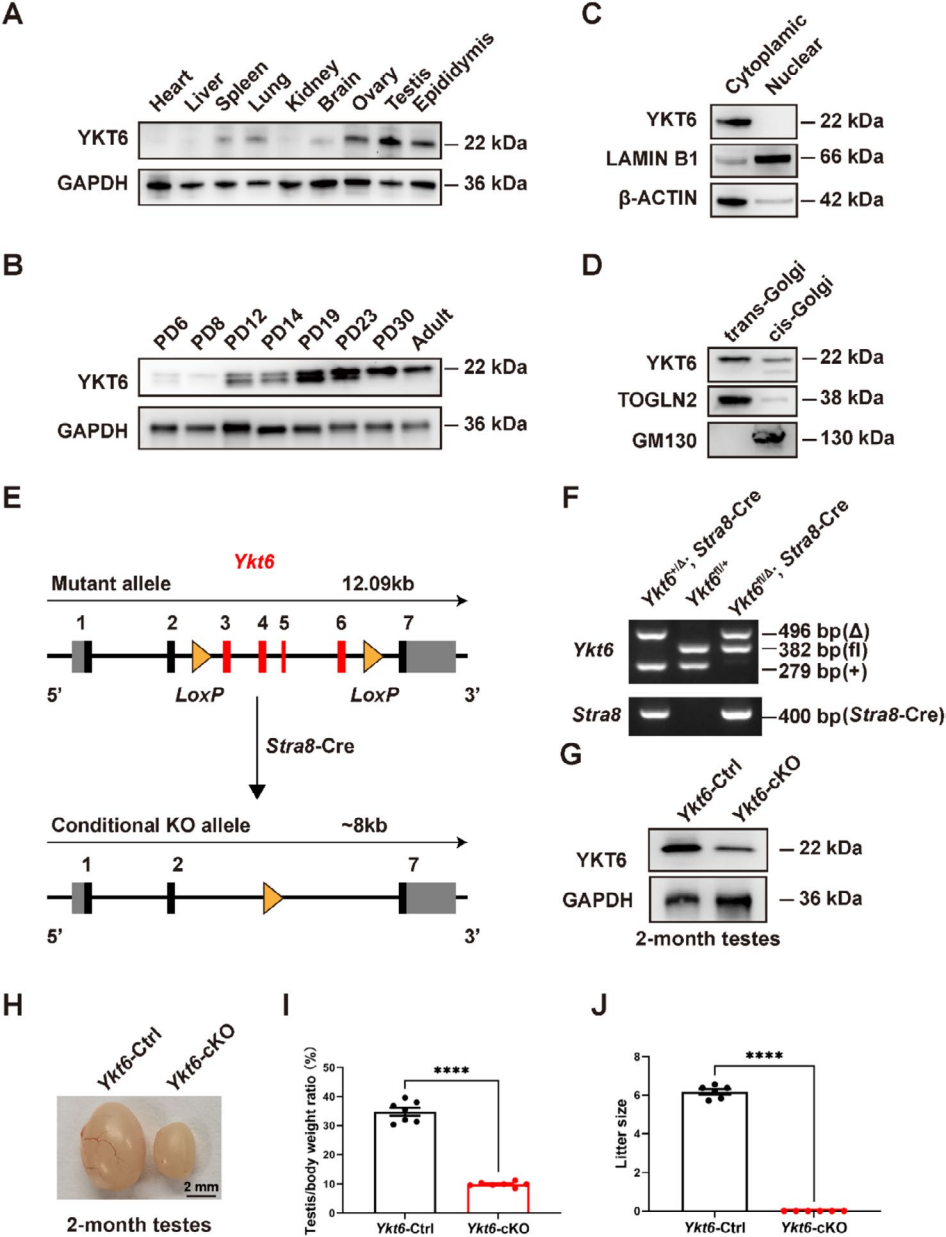
YKT6 is highly expressed in testes and is essential for fertility in male mice. **(A)** Western blots of YKT6 expression in different tissues from adult mice. GAPDH was used as the loading control. **(B)** Western blots of YKT6 expression in mouse testes from PD6 to adult mice. **(C)** Western blots of cytoplasmic and nuclear fractions of testes from adult mice. Lamin B1 served as a marker for the nuclear fraction; β‐actin was used as a marker for the cytoplasmic fraction. **(D)** Western blots of trans‐Golgi and cis‐Golgi fractions in testes from adult mice. TOGLN2 served as a marker of the trans‐Golgi fraction; GM130 served as a marker for the cis‐Golgi fraction. **(E)** Schematic of the strategy for conditional *Ykt6* knockout in testes. Grey boxes indicate non‐coding regions. Black and red boxes represent coding regions, with red boxes indicating the selectively ablated exons. Yellow arrowheads indicate *LoxP* insertion sites. **(F)** An example PCR confirming the different genotypes. **(G)** Western blots of YKT6 expression in *Ykt6*‐Ctrl and *Ykt6*‐cKO testes from adult mice. **(H)** Representative image of *Ykt6*‐Ctrl and *Ykt6*‐cKO testes from PD60 mice. **(I)** Quantitative analysis of the testis weight/body weight ratio in adult *Ykt6*‐Ctrl and *Ykt6*‐cKO mice. Columns show means ± SEM. ****p < 0.0001, Student's t‐test. **(J)** Litter size after mating of *Ykt6*‐Ctrl and *Ykt6*‐cKO males with wild‐type females. Columns display means ± SEM. ****p < 0.0001, Student's t‐test.

To explore the possible functions of YKT6 in male fertility, we first determined the spatial distribution of its protein expression across different tissues in C57BL/6J mice, including heart, liver, spleen, lung, kidney, brain, ovary, testis and epididymis. Western blots revealed that, among these tissues, YKT6 was expressed at the highest levels in reproductive organs, including testis and epididymis, of 2‐month‐old adult mice (Figure 1A). As the first round of spermatogenesis starts soon after birth and completes within the first 35 days of postnatal development in mice [34], we next sought to determine the stage of spermatogenic cells at which YKT6 is first expressed by Western blots of mouse testes sampled at postnatal days (PD) 6, 8, 12, 14, 19, 23, 30 and 2 months. This analysis showed that YKT6 protein levels peaked at PD12 and PD19 (Figure 1B), which was consistent with our observations of its elevated expression at zygotene and late diplotene stages in the spermatogenesis proteome [35] (Figure S1A), collectively suggesting that YKT6 might play a role in the process of spermatogenesis.

To observe the subcellular localization of YKT6 in mouse testes, we fractionated different cellular compartments of testes from 2‐month‐old adult mice. Subsequent Western blots indicated that YKT6 was only detectable in cytoplasmic and not nuclear fractions (Figure 1C). Furthermore, YKT6 mainly localised in the trans‐Golgi rather than cis‐Golgi fraction (Figure 1D). Immunofluorescent (IF) staining for YKT6 and the Golgi apparatus marker, GM130, in 293 T cells and human testis sections both showed that YKT6 was distributed throughout the cytoplasm and generally exhibited high signal intensity in regions where it co‐localised with GM130 (Figure S1B,C).

To then investigate the function of YKT6 in spermatogenesis, we mated *Ykt6*‐floxed mice, in which exons 3–6 were flanked by two *LoxP* sites, with *Stra8*‐Cre transgenic mice, which first express CRE recombinase at PD3, affecting germ cells starting from type A1 spermatogonia [36]. After confirming by PCR that *Ykt6* was indeed ablated or intact in the respective genomes of the *Ykt6* ^fl/Δ^; *Stra8*‐Cre (*Ykt6*‐cKO) progeny and their littermate controls (*Ykt6*‐Ctrl) (Figure 1F), Western blots further verified that YKT6 protein levels were reduced in *Ykt6*‐cKO testes (Figure 1G). These results indicated that we had successfully established a conditional knockout mouse line with *Ykt6* ablation specifically in pre‐meiotic germ cells and meiotic spermatocytes. Gross phenotype analysis showed that *Ykt6*‐cKO male mice had significantly smaller testes, as well as a lower testis‐to‐body weight ratio compared to their littermate controls at 2 months (Figure 1H,I). Over a 4‐week mating period, *Ykt6*‐cKO males sired no pups, compared to an average of six pups per litter with *Ykt6*‐Ctrl males, supporting a complete male sterility phenotype (Figure 1J).

**FIGURE 2:**
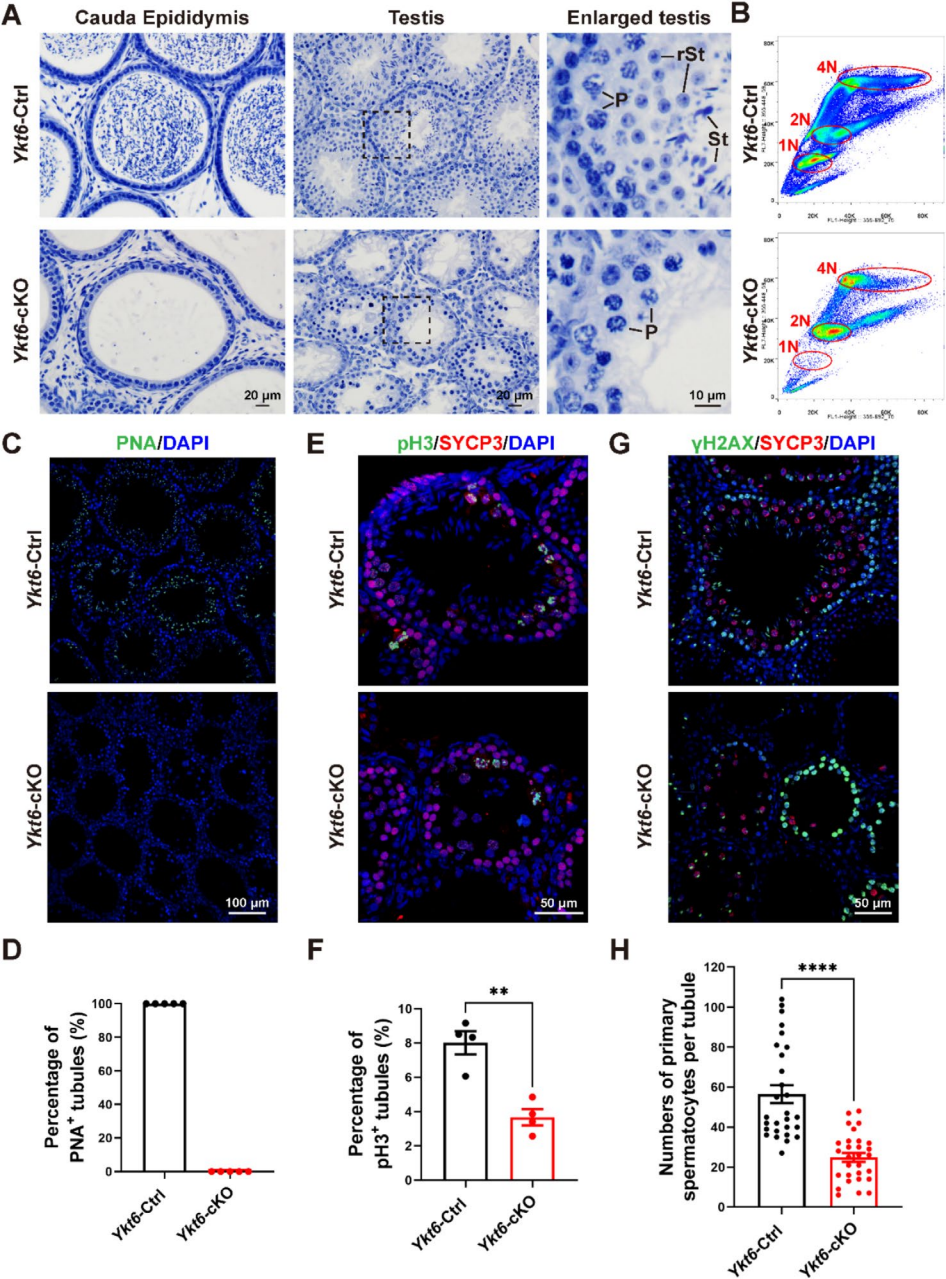
Spermatogenesis is disrupted in *Ykt6*‐cKO mice. **(A)** Haematoxylin staining of epididymis and testis sections from adult *Ykt6*‐Ctrl and *Ykt6*‐cKO mice. P, pachytene spermatocytes. rSt, round spermatids. St, spermatids. **(B)** Flow cytometry analysis of the spermatogenic population in *Ykt6*‐Ctrl and *Ykt6*‐cKO testes. Circle 1 N indicates the haploid (i.e., spermatid) population; circle 2 N indicates the diploid population (i.e., somatic cells and secondary spermatocytes); circle 4 N indicates the tetraploid (i.e., primary spermatocyte) population. **(C)** Immunofluorescent (IF) staining for the acrosome marker, PNA (green), in adult *Ykt6*‐Ctrl and *Ykt6*‐cKO testes. Nuclei were stained by DAPI. **(D)** Percentage of PNA^+^ tubules in adult *Ykt6*‐Ctrl and *Ykt6*‐cKO testes. **(E)** IF staining for pH 3 (green) and SYCP3 (red) in adult *Ykt6*‐Ctrl and *Ykt6*‐cKO testes. pH 3 served as a marker for condensed chromosomes during late G2 and metaphase; SYCP3 was used as a marker for the lateral element of the synaptonemal complex. **(F)** Percentage of pH 3^+^ tubules in *Ykt6*‐Ctrl and *Ykt6*‐cKO testes. Columns display means ± SEM. **p < 0.01, Student's t‐test. **(G)** IF staining for DNA double‐stranded break marker, γH2AX (green), and SYCP3 (red) in adult *Ykt6*‐Ctrl and *Ykt6*‐cKO testes. **(H)** Quantification of primary spermatocyte number per tubule in *Ykt6*‐Ctrl and *Ykt6*‐cKO testes. Columns display means ± SEM. ***p < 0.001, Student's t‐test.

Histological analysis of testes sections from 2‐month mice revealed that spermatids were absent in both epididymis tubules and seminiferous tubules of *Ykt6*‐cKO mice, but were abundant in corresponding samples of *Ykt6*‐Ctrl mice (Figure 2A). Further validation of these observed defects in spermatogenesis by flow cytometry revealed that haploid spermatids were completely absent, while late tetraploid spermatocytes were substantially reduced in the spermatogenic population of *Ykt6*‐cKO testes compared to the *Ykt6*‐Ctrl group (Figure 2B). In addition, IF staining for the acrosome marker, PNA, confirmed that no spermatids were present in *Ykt6*‐cKO testes (Figure 2C,D). Subsequent co‐staining for histone H3 Ser10 phosphorylation (pH 3), a marker of condensed chromosomes during late G2 and metaphase, and synaptonemal complex protein 3 (SYCP3), a marker of the lateral element of the synaptonemal complex, revealed that the number of tubules containing metaphase I spermatocytes was significantly reduced in *Ykt6*‐cKO testes (Figure 2E,F). Moreover, co‐staining for the DSB marker, γH2AX, with SYCP3 further indicated that the prophase I spermatocyte population was also obviously decreased in *Ykt6*‐cKO mice compared to that in *Ykt6*‐Ctrl testes (Figure 2G,H). Taken together, these findings suggested that YKT6 plays an essential role in mouse spermatogenesis, whereas its ablation results in the loss of spermatogenic cells and subsequent male infertility.

### 3.2 YKT6 Ablation Alters Spermatocyte Populations Without Affecting Meiotic Chromosomal Behaviours

Based on our observations of reduced prophase I spermatocyte populations in adult *Ykt6*‐cKO mice, we hypothesised that loss of *Ykt6* could lead to meiotic failure. We noted that *Ykt6*‐cKO male mice began to exhibit a reduced testis‐to‐body weight ratio at PD20 (Figure 2A–C). At the same time, haematoxylin and IF staining both showed that germ cell counts were significantly lower at PD20 in *Ykt6*‐cKO mice compared to that in *Ykt6*‐Ctrl mice (Figure 2D–H), supporting that *Ykt6* depletion disrupts the first round of meiosis.

**FIGURE 3:**
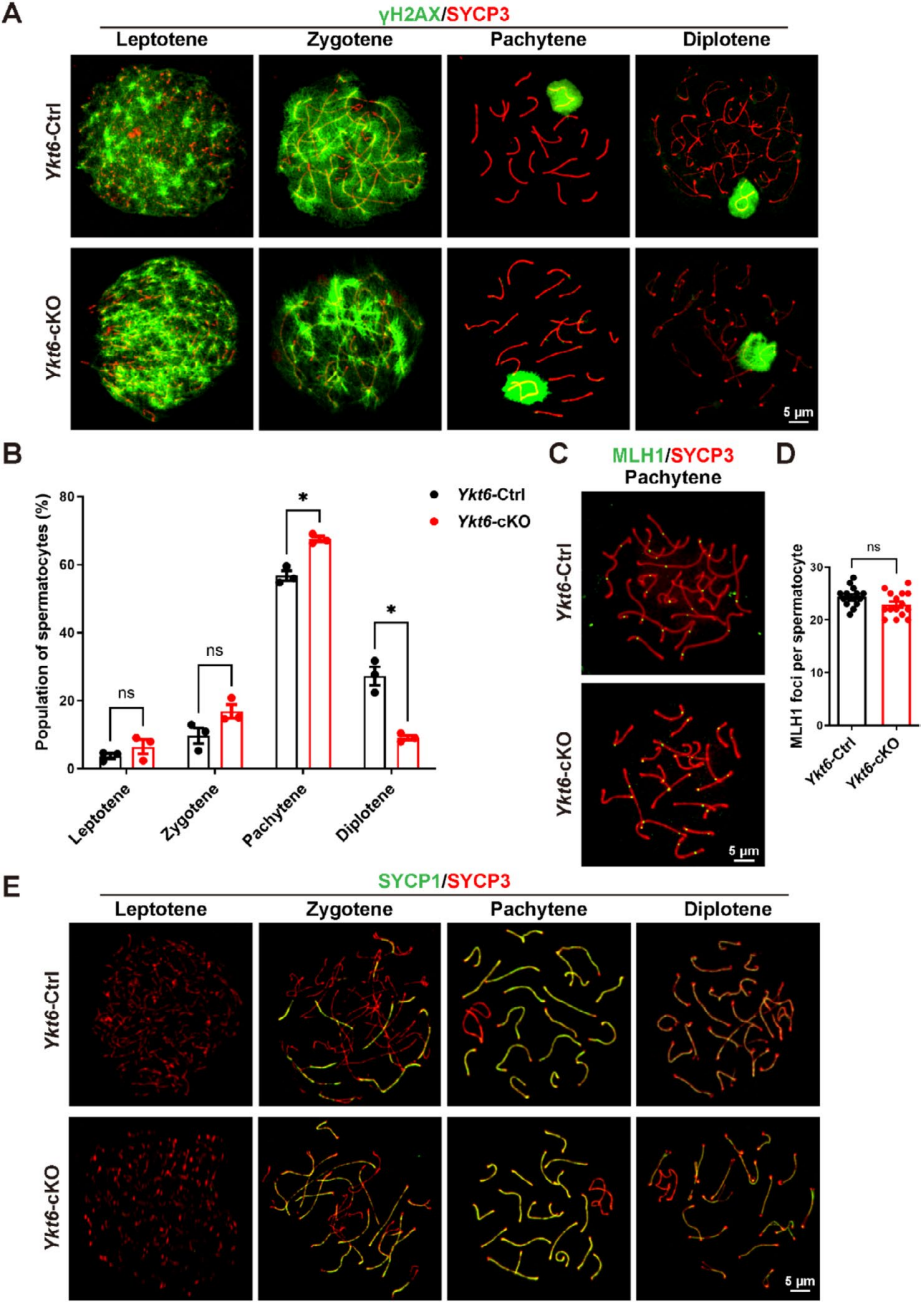
YKT6 ablation alters spermatocyte populations without affecting meiotic chromosomal behaviours. **(A)** IF staining for γH2AX (green) and SYCP3 (red) in chromosome spreads of *Ykt6*‐Ctrl and *Ykt6*‐cKO testicular cells. **(B)** Percentage of cells at successive stages of meiotic prophase I in chromosome spreads of spermatocytes in (A). Columns display means ± SEM. ns, *p* > 0.05, *p < 0.05, Student's t‐test. **(C)** IF staining for crossover recombination site marker, MLH1 (green), and SYCP3 (red) in chromosome spreads of *Ykt6*‐Ctrl and *Ykt6*‐cKO testicular cells. **(D)** Quantification of MLH1 foci number per spermatocyte. Columns display means ± SEM. ns, *p* > 0.05, Student's t‐test. **(E)** IF staining for the central element of the synaptonemal complex marker, SYCP1 (green), and SYCP3 (red) in chromosome spreads of *Ykt6*‐Ctrl and *Ykt6*‐cKO testicular cells.

To further explore events leading to this apparent failure of the meiotic process in *Ykt6*‐cKO male mice, we examined DSB formation by IF staining for γH2AX and SYCP3 in chromosome spreads of testes from 2‐month‐old mice, as γH2AX participates in DSB repair and is robustly expressed in the nucleus from leptotene to zygotene stages, and also enriched in the sex body (XY body) during the pachytene and diplotene stages [37]. We found no difference in γH2AX signal between *Ykt6*‐Ctrl and *Ykt6*‐cKO spermatocytes (Figure 3A), suggesting that DSB formation and repair both progressed normally during these stages in YKT6‐deficient spermatocytes. However, quantitative image analysis of spermatocytes in each stage indicated that pachytene populations were significantly higher in *Ykt6*‐cKO mice, while diplotene populations were decreased compared with their proportions in the *Ykt6*‐Ctrl group (Figure 3B). Examination of the crossover recombination marker protein, MLH1, by IF staining showed that YKT6‐deficient pachytene spermatocytes had a comparable number of MLH1 foci to that in control spermatocytes (Figure 3C,D), suggesting that crossover recombination was complete in *Ykt6*‐cKO mice. To then test whether assembly and disassembly of the synaptonemal complex proceeded normally, we monitored the distribution of SYCP1, as co‐labeling for SYCP3 and SYCP1 can highlight regions of synapsis in spermatocytes [4]. This analysis similarly showed no difference in synaptonemal complex formation or dissolution between *Ykt6*‐Ctrl and *Ykt6*‐cKO mice (Figure 3E). These results suggested that YKT6 deficiency disturbs the population of spermatocytes but does not affect chromosomal behaviours during meiosis.

### 3.3 *Ykt6*‐cKO Testes Contain Increased Syncytia in Seminiferous Tubules and Abnormal Golgi Apparatus and Lysosomes in Spermatocytes

**FIGURE 4:**
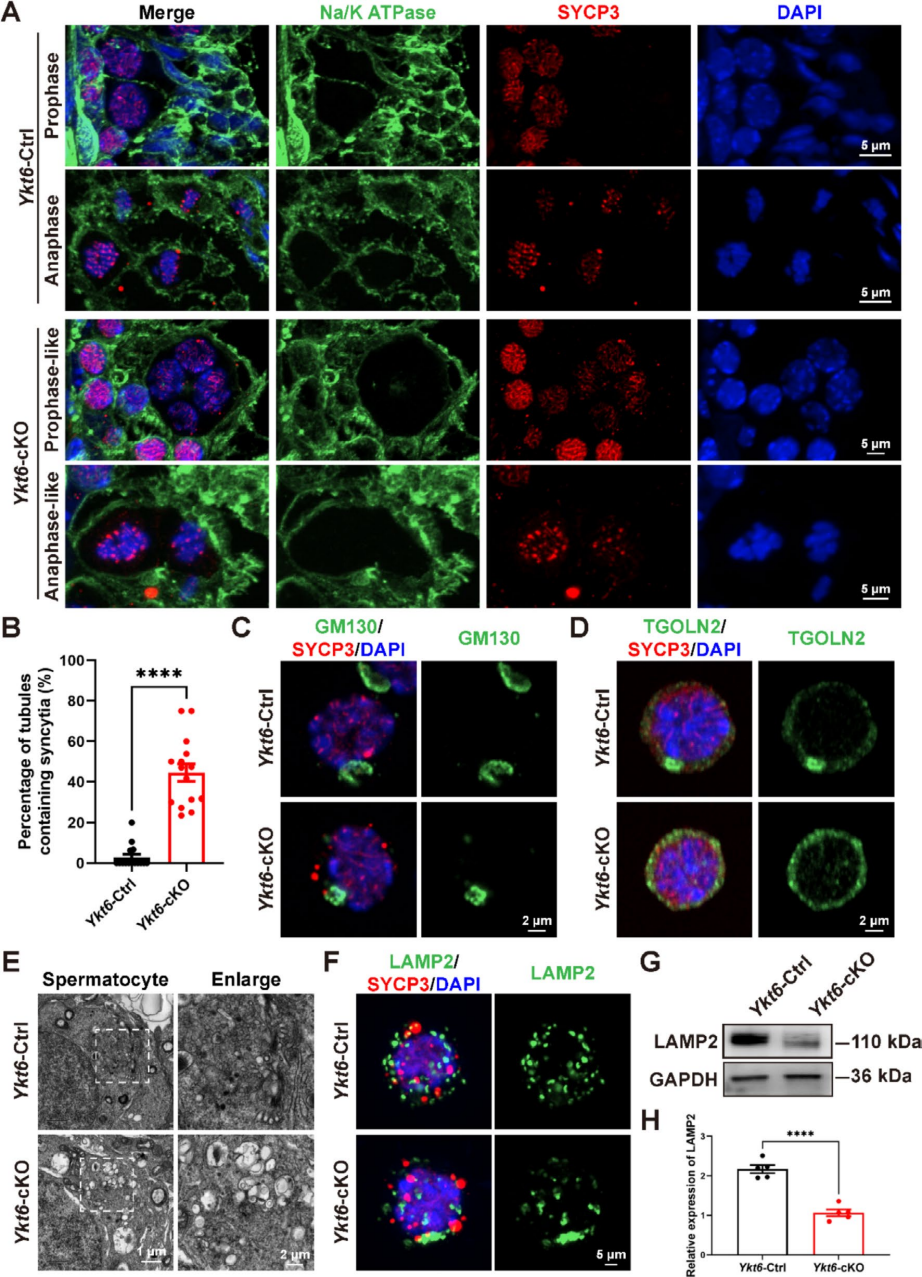
YKT6 deficiency leads to increased syncytia in seminiferous tubules and abnormal Golgi morphology and lysosome formation in spermatocytes. **(A)** IF staining for the cell membrane marker, Na/K ATPase (green), and SYCP3 (red) in adult *Ykt6*‐Ctrl and *Ykt6*‐cKO testes. **(B)** Percentage of tubules containing syncytia in adult *Ykt6*‐Ctrl and *Ykt6*‐cKO testes. Syncytia were detected in 2.90 ± 1.49% of seminiferous tubules of *Ykt6*‐Ctrl testes, whereas 44.59% ± 4.33% of *Ykt6*‐cKO tubules contained syncytia. Columns display means ± SEM. ****p < 0.0001, Student's t‐test. **(C)** IF staining for GM130 (green) and SYCP3 (red) in *Ykt6*‐Ctrl and *Ykt6*‐cKO testicular cells. **(D)** IF staining for TOGLN2 (green) and SYCP3 (red) in *Ykt6*‐Ctrl and *Ykt6*‐cKO testicular cells. **(E)** Ultrastructural analysis of spermatocytes from *Ykt6*‐Ctrl and *Ykt6*‐cKO testes by TEM. **(F)** IF staining for lysosomal marker, LAMP2 (green), and SYCP3 (red) in *Ykt6*‐Ctrl and *Ykt6*‐cKO testicular cells. **(G)** Western blots of LAMP2 expression in *Ykt6*‐Ctrl and *Ykt6*‐cKO testes. **(H)** Relative LAMP2 expression in (G) normalised against GAPDH, the loading control. Columns display means ± SEM. ****p < 0.0001, Student's t‐test.

In light of our earlier findings, we next co‐stained for the cell membrane marker, Na/K ATPase, along with SYCP3, and noted the syncytia appeared more in the absence of *Ykt6*. Interestingly, we observed that 44.59% ± 4.33% of tubules in *Ykt6*‐cKO testes contained syncytia with two or more meiotic nuclei, ranging from prophase I to anaphase I, whereas syncytia were rarely observed in tubules of *Ykt6*‐Ctrl testes (2.90% ± 1.49% of tubules) (Figure 4A,B).

Considering the localization of YKT6 in the Golgi apparatus, we then examined the morphology of the Golgi apparatus by co‐staining for the cis‐Golgi marker, GM130, with SYCP3, which revealed that the lamellar structure of the cis‐Golgi apparatus was collapsed in YKT6‐deficient spermatocytes (Figure 4C). Additionally, IF co‐staining for the trans‐Golgi marker, TGOLN2, with SYCP3 showed that aggregative spots were absent in YKT6‐deficient spermatocytes, but obviously formed in *Ykt6*‐Ctrl spermatocytes (Figure 4D). Further examination by transmission electron microscopy (TEM) indicated that YKT6‐deficient spermatocytes lacked a normal Golgi apparatus and instead contained numerous large vacuoles (Figure 4E). Given that lysosomes originate in the Golgi apparatus, we next investigated lysosome formation by IF co‐staining for the lysosomal marker, LAMP2, with SYCP3 and observed that the lysosomal distribution was obviously aberrant in YKT6‐deficient spermatocytes compared to that in the *Ykt6*‐Ctrl group (Figure 4F). Additionally, Western blot detection of LAMP2 indicated that its relative protein expression was significantly reduced in *Ykt6*‐cKO testes (Figure 4G,H). These findings suggested that YKT6 deficiency leads to increased syncytia, as well as abnormal Golgi apparatus structures and lysosome distribution in spermatocytes.

### 3.4 YKT6 Deficiency Results in Dysregulated Vesicular Transport and Intercellular Bridges

**FIGURE 5:**
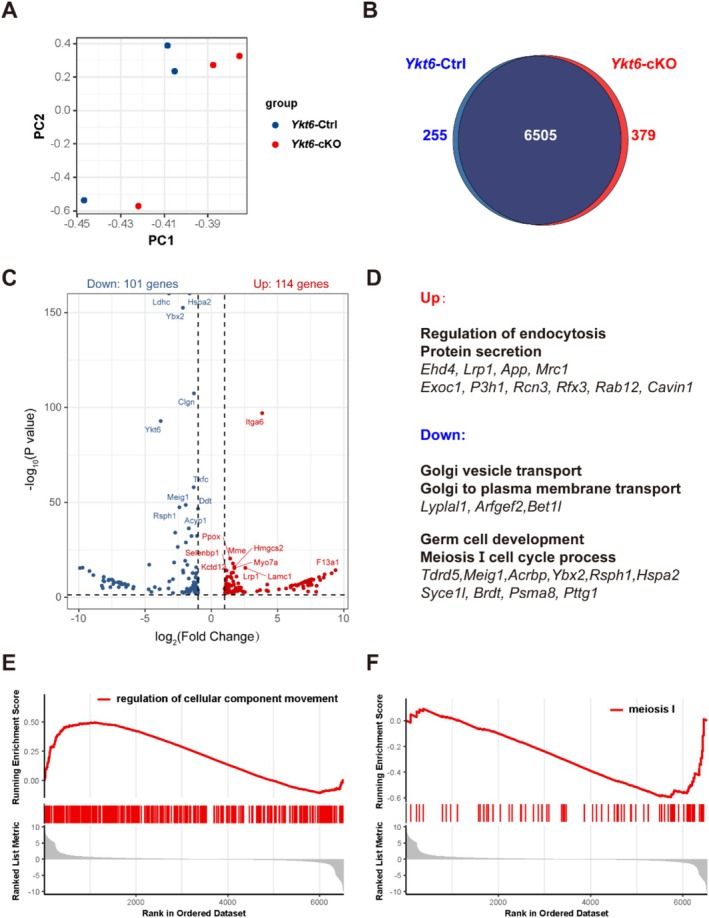
Quantitative proteomics analysis of spermatocytes. **(A)** PCA of proteomics data showing proteins with significantly differential abundance between *Ykt6*‐Ctrl and *Ykt6*‐cKO samples. **(B)** Venn diagram showing overlap of proteins identified in both *Ykt6*‐Ctrl and *Ykt6*‐cKO samples. **(C)** Volcano plot of significant differentially expressed proteins in the spermatocytes of *Ykt6*‐cKO mice compared with *Ykt6*‐Ctrl. Blue dots represent significantly downregulated proteins; red dots represent significantly upregulated proteins (|log_2_FC| > 1.0 and *p* < 0.05). **(D)** GO enrichment analysis of upregulated and downregulated proteins in spermatocytes of *Ykt6*‐cKO mice compared with *Ykt6*‐Ctrl. Representative pathways and genes in up‐ and downregulated groups are shown. **(E‐F)** GSEA of differentially expressed proteins in spermatocytes of *Ykt6*‐cKO mice compared with *Ykt6*‐Ctrl samples.

Considering the abnormalities we observed in the Golgi apparatus and lysosome formation in YKT6‐deficient spermatocytes, we hypothesised that loss of YKT6 function might disrupt protein homeostasis. To test this possibility, we conducted a quantitative proteomic analysis in tetraploid spermatocytes obtained by fluorescence‐activated cell sorting (FACS). PCA showed an obvious separation of samples between *Ykt6*‐cKO and *Ykt6*‐Ctrl samples (Figure 5A). Subsequent analysis of individual proteins identified 6505 proteins common to both groups, while 379 proteins were exclusively detected in *Ykt6*‐cKO samples and 255 proteins were unique to *Ykt6*‐Ctrl samples (Figure 5B). DESeq2 analysis of differentially enriched proteins indicated that 114 proteins were differentially upregulated, while 101 proteins were significantly downregulated in *Ykt6*‐cKO samples (Figure 5C). Notably, GO analysis of differentially expressed proteins revealed that upregulated proteins were associated with regulation of endocytosis and protein secretion, while downregulated proteins were enriched in processes such as Golgi vesicle transport, Golgi to plasma membrane transport, germ cell development and meiosis I cell cycle (Figure 5D). Similarly, GO analysis of proteins found only in *Ykt6*‐cKO samples showed enrichment for terms such as vacuolar membrane and early endosome, whereas proteins unique to *Ykt6*‐Ctrl samples were enriched in terms related to Golgi apparatus subcompartment and trans‐Golgi network (Figure 3A,B). Additionally, GSEA indicated that proteins associated with regulation of cellular component movement were globally upregulated, while proteins associated with meiosis I were globally downregulated in *Ykt6*‐cKO samples (Figure 5E,F).

**FIGURE 6:**
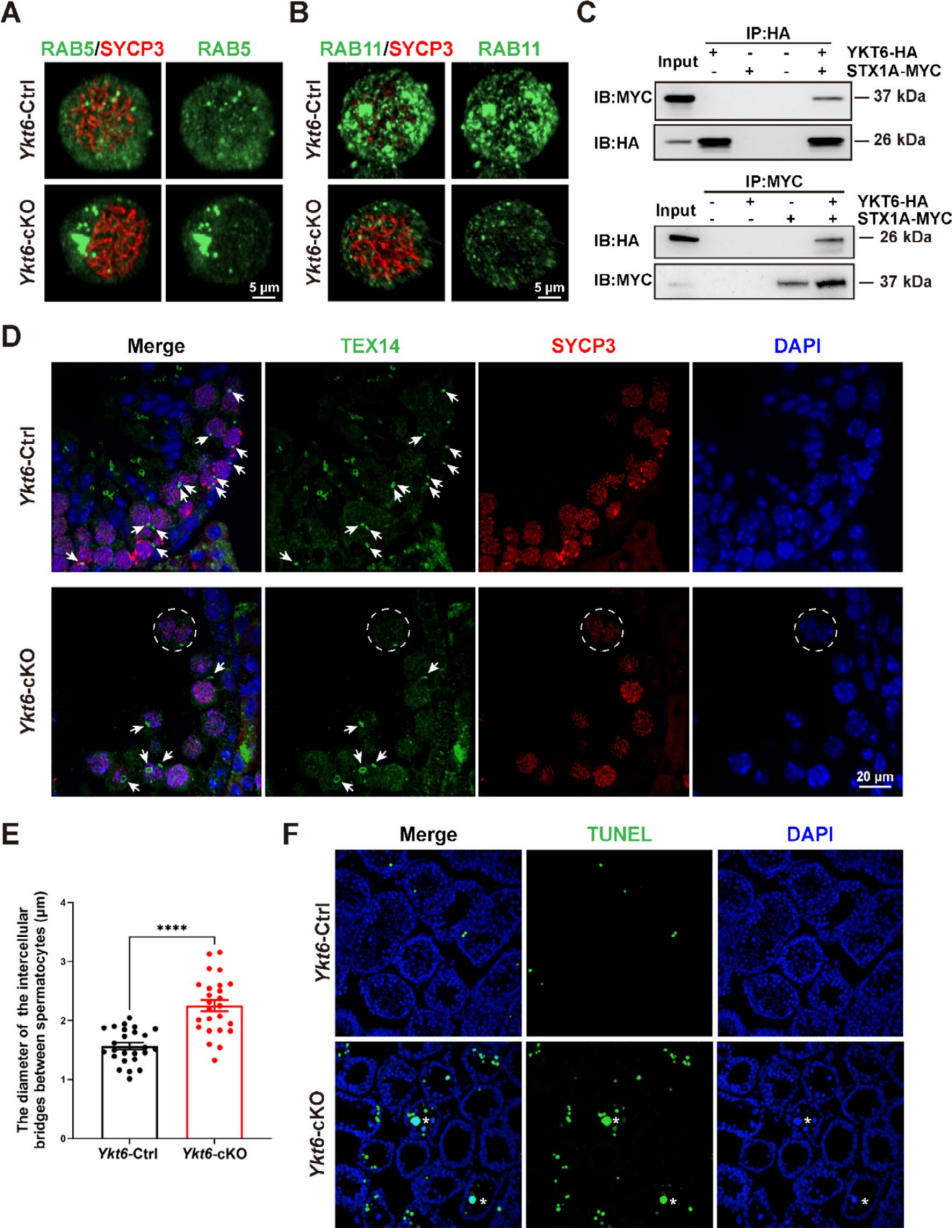
YKT6 deficiency results in dysregulated vesicular transport and aberrantly extended intercellular bridges of spermatocytes. **(A)** IF staining for early endosome marker, RAB5 (green), and SYCP3 (red) in *Ykt6*‐Ctrl and *Ykt6*‐cKO testicular cells. **(B)** IF staining for recycling endosome marker, RAB11 (green), and SYCP3 (red) in *Ykt6*‐Ctrl and *Ykt6*‐cKO testicular cells. **(C)** Co‐immunoprecipitation of YKT6‐STX1A in vitro. HA‐tagged mouse YKT6 and MYC‐tagged mouse STX1A were co‐expressed in HEK293T cells. pCDNA3.1–3 × HA‐C and pCDNA3.1–3 × Myc‐C vectors were used as negative controls. **(D)** IF staining for intercellular bridge marker, TEX14 (green), and SYCP3 (red) in *Ykt6*‐Ctrl and *Ykt6*‐cKO testes. Arrows point to intercellular bridges between spermatocytes; circles indicate syncytia. **(E)** Quantitative analysis of intercellular bridge diameter between spermatocytes in *Ykt6*‐Ctrl and *Ykt6*‐cKO testes. Columns display means ± SEM. ****p < 0.0001, Student's t‐test. **(F)** TUNEL staining of seminiferous tubules in adult *Ykt6*‐Ctrl and *Ykt6*‐cKO testes. * indicates seminiferous tubules containing syncytia.

Given these findings of dysregulated protein expression in multiple vesicular transport pathways under conditional *Ykt6* knockout, we conducted IF staining for SYCP3 along with the early endosome marker, RAB5, and observed that the RAB5 signal was significantly stronger in YKT6‐deficient spermatocytes (Figure 6A), indicating obvious accumulation of early endosomes. Alternatively, co‐staining for SYCP3 with the recycling endosome marker, RAB11, showed a concomitant decrease in the distribution of recycling endosomes (Figure 6B). These results suggested that vesicular transport was dysregulated in YKT6‐deficient spermatocytes. As YKT6 has been shown to interact with Syntaxin 1 (STX1) during the fusion of constitutive secretory carriers with the plasma membrane in *Drosophila* [19], we conducted co‐immunoprecipitation assays in lysates of 293 T cells co‐expressing the recombinant mouse proteins, HA::YKT6 and MYC::STX1A and found that these proteins could mutually precipitate each other (Figure 6C). IF staining for STX1A and GM130 in 293 T cells further showed that STX1A exhibited high signal intensity in the Golgi region, similar to YKT6 localization patterns (Figure S1D). These results indicated that mouse YKT6 could interact with STX1A to form a complex in vitro.

Based on prior knowledge that the diameter of intercellular bridges increases within a specific range as germ cells differentiate (starting at ~1.0–1.3 μm in spermatogonia, expanding to 1.4–1.7 μm spermatocytes, and reaching ≥ 1.8 μm in spermatids), and that the membrane origin of intercellular bridges requires vesicular transport [10, 38], we therefore examined intercellular bridges by IF staining for the intercellular bridge marker, TEX14, with SYCP3. This analysis showed that intercellular bridge structures were absent within syncytia. Moreover, the diameter of TEX14 rings, representing intercellular bridge diameter between spermatocytes, was significantly greater in *Ykt6*‐cKO testes compared to that in *Ykt6*‐Ctrl testes (Figure 6D,E). This aberrant enlargement suggested structural dysregulation in the formation of intercellular bridges between spermatocytes. At the same time, TUNEL staining indicated that apoptotic signals were elevated in both syncytia and non‐syncytia of these *Ykt6*‐cKO seminiferous tubules relative to that in *Ykt6*‐Ctrl samples (Figure 6F).

Overall, these results suggested that YKT6 plays an essential role in spermatogenesis, likely through interactions with STX1A to regulate vesicular transport, resulting in stabilisation of intercellular bridges between spermatocytes.

## 4 Discussion

In this study, we identified the SNARE family member, YKT6, which functions in several vesicular transport pathways, as an essential protein for successful meiosis during spermatogenesis in mice. YKT6 is highly expressed in the testes of adult mice, with its highest protein accumulation detected at PD12 and PD19, corresponding to the zygotene and late diplotene stages of meiosis. Proteins associated with vesicle trafficking typically function during the later stages of spermatogenesis. For instance, RAB GTPases are associated with cytoskeletal organisation, Golgi‐mediated acrosome formation, and changes in sperm morphology during spermiogenesis, all of which occur after meiosis [39]. Here, specific ablation of *Ykt6* in pre‐meiotic germ cells and meiotic spermatocytes led to complete sterility and meiotic arrest in male mice.

To ensure the successful completion of meiosis, spermatocytes undergo a tightly coordinated series of events, including the formation of DSBs, synapsis, crossover recombination and synaptonemal complex disassembly [3]. Although spermatocyte populations were disturbed in *Ykt6*‐cKO testes, no aberrant behaviour was observed among meiotic chromosomes. YKT6‐deficient spermatocytes undergo normal DSB formation and repair, crossover recombination and assembly/disassembly of the synaptonemal complex.

Consistent with previous reports [40], we found that YKT6 accumulates exclusively in the cytoplasm of testicular cells in mice, where it largely co‐localises with the Golgi apparatus. Having ruled out anomalies in chromosomal events during meiosis, we noted an increase in the abundance of syncytia along with abnormal Golgi morphology in *Ykt6*‐cKO testes. Our results showed that syncytia contained two or more meiotic nuclei ranging from prophase I to anaphase I in *Ykt6*‐cKO testes. At the same time, we observed a collapsed lamellar structure of the cis‐Golgi apparatus and loss of immunofluorescent signal for aggregative spots of the trans‐Golgi apparatus in YKT6‐deficient spermatocytes. Further observations showed that Golgi‐derived lysosomes are also abnormally distributed and relatively sparse in *Ykt6*‐cKO testes. These findings suggested disruption of Golgi‐associated functions, which could plausibly disturb protein homeostasis in YKT6‐deficient spermatocytes. Exploring this possibility, we performed quantitative proteomic profiling of tetraploid spermatocytes obtained from *Ykt6‐*Ctrl and *Ykt6*‐cKO mice at PD16, which identified 114 significantly upregulated proteins enriched in regulation of endocytosis, and 101 downregulated proteins enriched in germ cell development in *Ykt6*‐cKO samples. Additionally, we found that proteins associated with regulation of cellular component movement were globally upregulated, while proteins associated with meiosis I were globally downregulated in *Ykt6*‐cKO samples, supporting a likely role of YKT6 in vesicular transport of spermatocytes. Further IF staining showed that early endosomes increasingly accumulate while recycling endosomes are depleted in YKT6‐deficient spermatocytes, thus confirming that *Ykt6* ablation causes dysregulation of vesicular transport.

The constitutive secretion pathway delivers lipids to the cell surface and is essential for cell growth and viability [19]. Cytokinesis in somatic cells includes the accumulation of membrane vesicles in the furrow and intercellular bridge, and concludes with the formation of a midbody, which is subsequently abscised to form individual daughter cells [41, 42]. In contrast, cytokinesis in germ cells results in the formation of a permanent intercellular bridge connecting the daughter cells through a large cytoplasmic channel [12]. TEX14 and other proteins come together to prevent abscission and form stable intercellular bridges [43]. Targeted deletion of *Tex14* disrupts intercellular bridges in all germ cells and causes male sterility [12]. Some proposed functions of the intercellular bridge in spermatogenesis include communication and synchronisation among germ cells and chromosome dosage compensation in haploid cells [10, 12]. While extensive studies have elucidated the critical role of vesicle transport in the formation of intercellular bridges during mitotic cytokinesis, the molecular mechanisms regulating analogous membrane remodelling events during meiosis remain scarcely explored. In germ cell development, vesicular transport to the plasma membrane is essential for maintaining and expanding intercellular bridges within a tightly controlled size range. STX2 functions in transporting seminolipids to the cell membrane, and its mutation is known to cause infertility strictly in males due to syncytial multinucleation of spermatogenic cells during the prophase of meiosis [38]. EXOC1 and SNAP23 interact with STX2, and deletion of these genes results in a similar phenotype [44]. Additionally, YKT6 has been shown to function coordinately with STX1A in secretory carrier fusion with the plasma membrane, and this process is conserved between yeast and humans [19]. Our results verified that murine YKT6 can interact with STX1A in vitro. In the absence of YKT6, spermatocytes exhibit an increase in the diameter of intercellular bridges, while intercellular bridges between tetraploid nuclei within syncytia vanish, thus revealing a potential role of YKT6 in stabilising intercellular bridges. Finally, we found that apoptotic signalling is elevated in both syncytia and non‐syncytia in seminiferous tubules of *Ykt6*‐cKO mice relative to that in *Ykt6*‐Ctrl samples.

Unfortunately, we found that none of the available antibodies targeting YKT6 were sufficiently specific for immunofluorescence assays in mice. Due to these limitations of immunofluorescent antibodies targeting mouse YKT6, we were unable to observe YKT6‐mediated vesicular transport in defective spermatocytes. In future studies, the development of effective YKT6 antibodies, or in vitro culture and transfection of spermatocytes, will enable real‐time observation of vesicular transport activities in spermatocytes.

In conclusion, this study shows that YKT6 is essential for spermatogenesis and male fertility in mice, potentially through its interactions with STX1A in mediating transport vesicle fusion to the plasma membrane to stabilise intercellular bridges between spermatocytes, and not by affecting chromosomal behaviour during meiosis. These results expand our understanding of YKT6 functions in spermatogenesis, and suggest possible targets for future interventions for male infertility.

## Author Contributions

H.L., Y.C. and G.F. designed the research and supervised the project. J.C., X.Y. and Z.W. performed the experimental operation and collected the data. J.C. designed the experiments, analysed the data, wrote the first draft of the manuscript and further edited upon input from all co‐authors. W.L., J.X., and Q.F. revised and discussed the manuscript. All authors have read and agreed to the published version of the manuscript.

## Conflicts of Interest

The authors declare no conflicts of interest.

## Supporting information


**Figure S1.** YKT6 is highly expressed during spermatogenesis and co‐localises with Golgi apparatus.
(A) Graph of changes in YKT6 expression across different stages of spermatogenesis from the mouse proteome [35]. Aun, Type A undifferentiated spermatogonia. eLL, early leptotene and leptotene. Z, zygotene. eP, early pachytene. mP, middle pachytene. lP, late pachytene. eD, early diplotene. lD, late diplotene. RS, round spermatid. (B) IF staining for YKT6 (green) and GM130 (red) in 293 T cell lines. (C) IF staining for YKT6 (green) and GM130 (red) in human testis sections. (D) IF staining for STX1A (green) and GM130 (red) in 293 T cell lines.
**Figure S2.** *Ykt6* depletion leads to impaired first round of meiosis.
(A–C) Quantitative analysis of testis weight/body weight ratios in *Ykt6*‐Ctrl and *Ykt6*‐cKO mice at PD14 (A), PD16 (B) and PD20 (C). Columns display means ± SEM. ns, *p* > 0.05, *p < 0.05, Student’s t‐test. (D–F) Haematoxylin staining of testes sections from *Ykt6*‐Ctrl and *Ykt6*‐cKO mice at PD14 (D), PD16 (E) and PD20 (F). (G) IF staining for germ cell marker, MVH (red), and Sertoli cell marker, SOX9 (green) in *Ykt6*‐Ctrl and *Ykt6*‐cKO testes at PD20. (H) Quantitative analysis of germ cell number per Sertoli cell in *Ykt6*‐Ctrl and *Ykt6*‐cKO testes. Columns display means ± SEM. **p < 0.01, Student’s t‐test.
**Figure S3.** GO enrichment analysis for proteins identified by quantitative proteomics analysis of spermatocytes.
(A) GO enrichment analysis of proteins detected only in *Ykt6*‐cKO samples. (B) GO enrichment analysis of proteins detected only in *Ykt6*‐Ctrl samples.

## Data Availability

The mass spectrometry proteomics data have been deposited to the ProteomeXchange Consortium (https://proteomecentral.proteomexchange.org) via the iProX partner repository [45, 46] with the dataset identifier PXD063213.
