# Supplementary material for: YKT6 Is Essential for Male Fertility by Promoting Meiosis Progression During Spermatogenesis of Mice
Source: Cell Prolif. 2025 Jun 18;59(1):e70079. doi: 10.1111/cpr.70079 (PMC12774620; doi:10.1111/cpr.70079)
Supplement: Supplementary file 1 — Figure S1. YKT6 is highly expressed during spermatogenesis and co‐localises with Golgi apparatus. (A) Graph of changes in YKT6 expression across different stages of spermatogenesis from the mouse proteome [35]. Aun, Type A undifferentiated spermatogonia. eLL, early leptotene and leptotene. Z, zygotene. eP, early pachytene. mP, middle pachytene. lP, late pachytene. eD, early diplotene. lD, late diplotene. RS, round spermatid. (B) IF staining for YKT6 (green) and GM130 (red) in 293 T cell lines. (C) IF staining for YKT6 (green) and GM130 (red) in human testis sections. (D) IF staining for STX1A (green) and GM130 (red) in 293 T cell lines. Figure S2. Ykt6 depletion leads to impaired first round of meiosis. (A–C) Quantitative analysis of testis weight/body weight ratios in Ykt6‐Ctrl and Ykt6‐cKO mice at PD14 (A), PD16 (B) and PD20 (C). Columns display means ± SEM. ns, p > 0.05, *p < 0.05, Student’s t‐test. (D–F) Haematoxylin staining of testes sections from Ykt6‐Ctrl and Ykt6‐cKO mice at PD14 (D), PD16 (E) and PD20 (F). (G) IF staining for germ cell marker, MVH (red), and Sertoli cell marker, SOX9 (green) in Ykt6‐Ctrl and Ykt6‐cKO testes at PD20. (H) Quantitative analysis of germ cell number per Sertoli cell in Ykt6‐Ctrl and Ykt6‐cKO testes. Columns display means ± SEM. **p < 0.01, Student’s t‐test. Figure S3. GO enrichment analysis for proteins identified by quantitative proteomics analysis of spermatocytes. (A) GO enrichment analysis of proteins detected only in Ykt6‐cKO samples. (B) GO enrichment analysis of proteins detected only in Ykt6‐Ctrl samples. [file CPR-59-e70079-s001.docx]

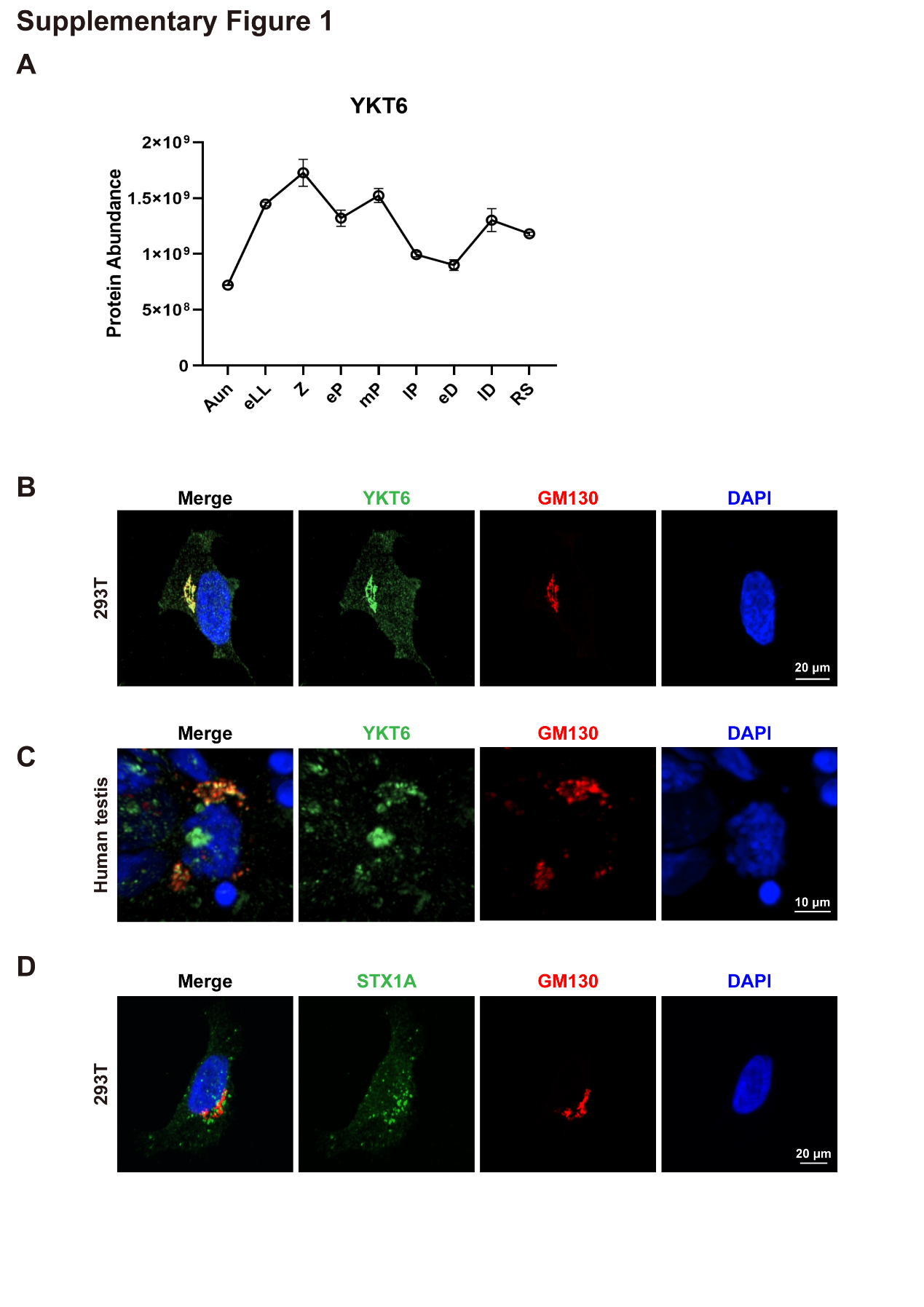


**Supplementary Figure 1.** YKT6 is highly expressed during spermatogenesis and co-localizes with Golgi apparatus.

**(A**) Graph of changes in YKT6 expression across different stages of spermatogenesis from the mouse proteome^35^. Aun, Type A undifferentiated spermatogonia. eLL, early leptotene and leptotene. Z, zygotene. eP, early pachytene. mP, middle pachytene. lP, late pachytene. eD, early diplotene. lD, late diplotene. RS, round spermatid. **(B)** IF staining for YKT6 (green) and GM130 (red) in 293T cell lines. **(C)** IF staining for YKT6 (green) and GM130 (red) in human testis sections. **(D)** IF staining for STX1A (green) and GM130 (red) in 293T cell lines.


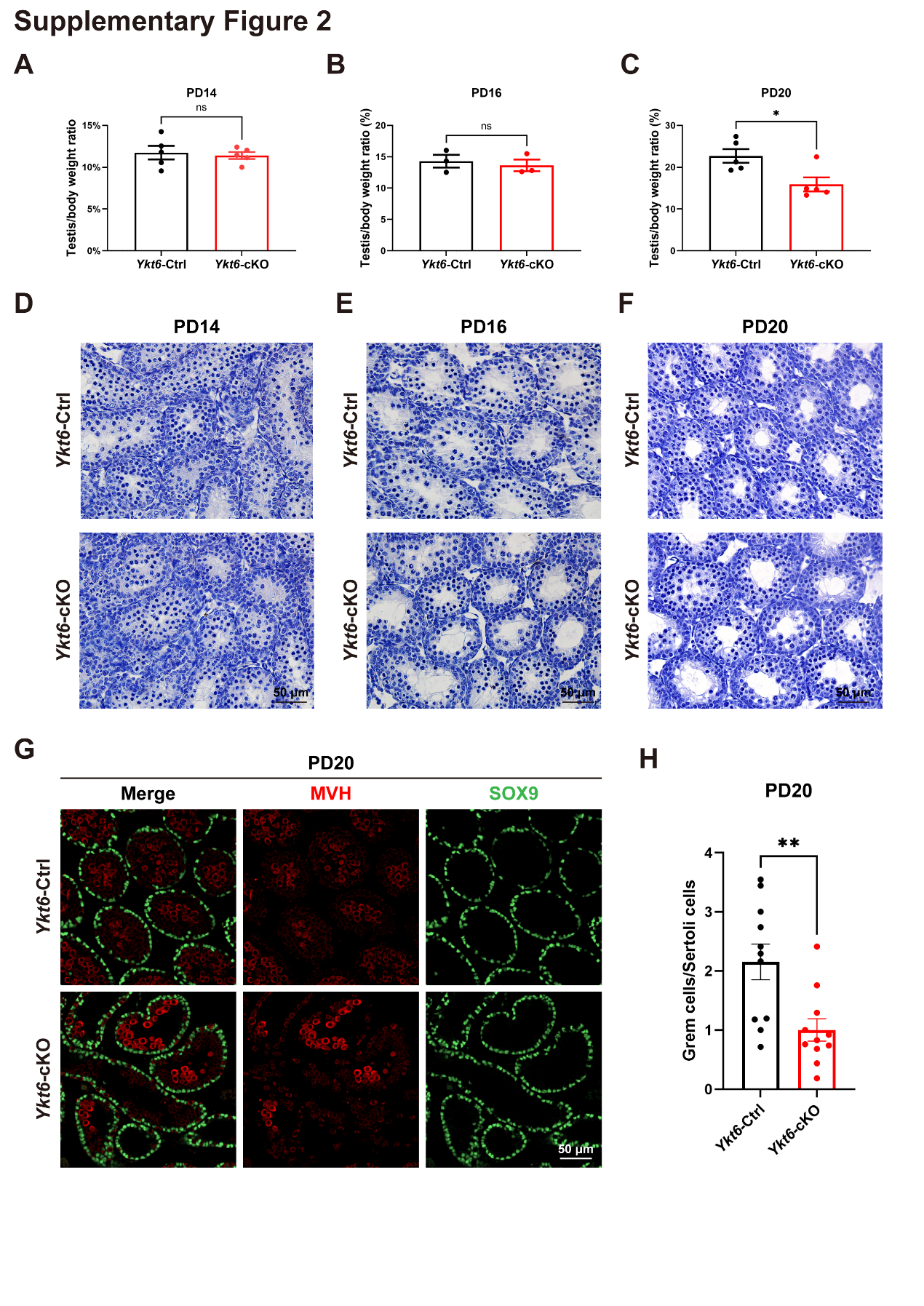


**Supplementary Figure 2.** *Ykt6* depletion leads to impaired first round of meiosis.

**(A-C)** Quantitative analysis of testis weight/body weight ratios in *Ykt6*-Ctrl and *Ykt6*-cKO mice at PD14 (**A**), PD16 (**B**), and PD20 (**C**). Columns display means ± SEM. ns, *P* > 0.05, **P* < 0.05, Student’s t-test. **(D-F)** Hematoxylin staining of testes sections from *Ykt6*-Ctrl and *Ykt6*-cKO mice at PD14 (**D**), PD16 (**E**), and PD20 (**F**). **(G)** IF staining for germ cell marker, MVH (red), and Sertoli cell marker, SOX9 (green) in *Ykt6*-Ctrl and *Ykt6*-cKO testes at PD20. **(H)** Quantitative analysis of germ cell number per Sertoli cell in *Ykt6*-Ctrl and *Ykt6*-cKO testes. Columns display means ± SEM. ***P* < 0.01, Student’s t-test.


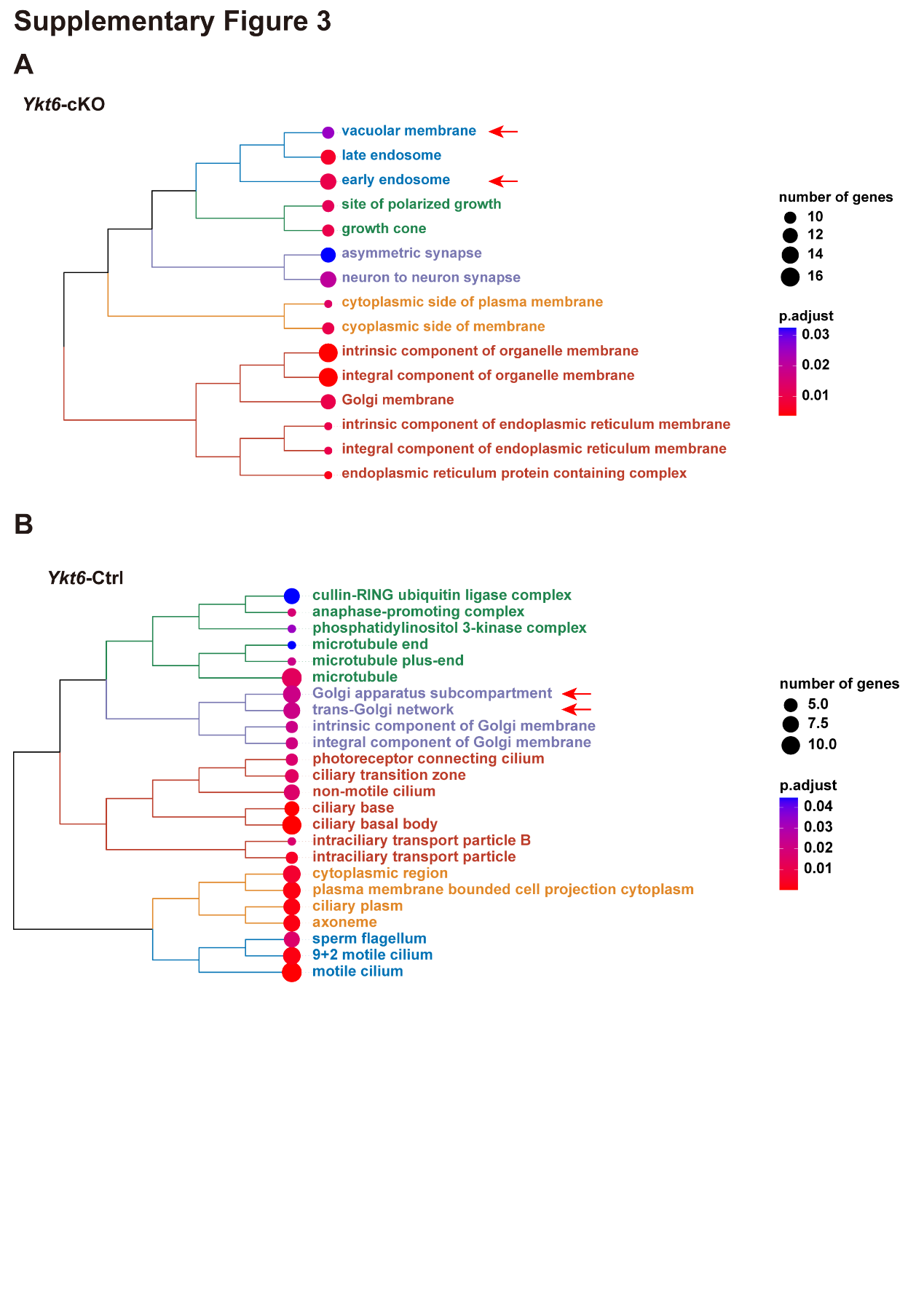


**Supplementary Figure 3.** GO enrichment analysis for proteins identified by quantitative proteomics analysis of spermatocytes.

**(A)** GO enrichment analysis of proteins detected only in *Ykt6*-cKO samples. **(B)** GO enrichment analysis of proteins detected only in *Ykt6*-Ctrl samples.
